# Constructing Stiff β-Sheet for Self-Reinforced Alginate Fibers

**DOI:** 10.3390/ma17133047

**Published:** 2024-06-21

**Authors:** Xuelai Xie, Min Cui, Tianyuan Wang, Jinhong Yang, Wenli Li, Kai Wang, Min Lin

**Affiliations:** 1 State Key Laboratory of Bio-Fibers and Eco-Textiles, College of Materials Science and Engineering, Shandong Collaborative Innovation Center of Marine Biobased Fibers and Ecological Textiles, Qingdao University, Qingdao 266071, China; 2 Institute of Flexible Electronics (IFE), Northwestern Polytechnical University (NPU), Xi’an 710072, China

**Keywords:** self-reinforcement, β-sheet, degradability, alginate fibers, polysaccharides

## Abstract

The application of alginate fibers is limited by relatively low mechanical properties. Herein, a self-reinforcing strategy inspired by nature is proposed to fabricate alginate fibers with minimal changes in the wet-spinning process. By adapting a coagulation bath composing of CaCl_2_ and ethanol, the secondary structure of sodium alginate (SA) was regulated during the fibrous formation. Ethanol mainly increased the content of β-sheet in SA. Rheological analysis revealed a reinforcing mechanism of stiff β-sheet for enhanced modulus and strength. In combination with Ca^2+^ crosslinking, the self-reinforced alginate fibers exhibited an increment of 39.0% in tensile strength and 71.9% in toughness. This work provides fundamental understanding for β-sheet structures in polysaccharides and a subsequent self-reinforcing mechanism. It is significant for synthesizing strong and tough materials. The self-reinforcing strategy involved no extra additives and preserved the degradability of the alginate. The reinforced alginate fibers exhibited promising potentials for biological applications.

## 1. Introduction

Sodium alginate (SA) is a naturally abundant polysaccharide derived from brown seaweeds. After extensive studies on biocompatibility, it has been proven non-toxic and non-immunogenic for various biomedical applications, such as wound healing [1,2], drug delivery [3], tissue engineering [4,5], and regenerative medicine [6]. An important feature of SA is its ability to crosslink with divalent cations like calcium ions due to rich carboxylate groups from repeating units of D-mannuronic acid (M units) and L-guluronic acid (G units). Kim et al. designed in situ aneurysm embolization to trigger tumor tissue necrosis under efficient coordination of SA with serum calcium [7]. Based on similar principles, alginate fibers can be produced through a sustainable and green wet-spinning process in aqueous solutions [8]. But the obtained fibers usually have lower tensile strength compared with other synthetic fibers, which limits their applications. Industrial production also requires a stable supply of high-quality and high molecular weight (*M_w_*) SA from aged seaweeds grown in limited areas, leading to extremely high costs. The demands for efficient reinforcing strategies are highlighted to produce alginate fibers of improved mechanical performance [9,10].

In fibrous reinforcement, a common strategy is to add second phase additives. The additives are stiff units such as Ag Nanowires [11], graphene oxide [12,13], carbon nanotubes [14], silica, etc., which increases the modulus and strength of fibers [15,16]. Similarly, compositing SA with other polymers such as chitosan, gelatin, and polyethylene glycol can also improve mechanical properties due to forming composite dissipation networks [17,18,19]. For example, Yu et al. reported that bacteria cellulose nanofibril could reinforce the tensile strength of alginate fibers by ~2 times [20]. Twisting the composite alginate fibers into a helical structure could achieve further enhancement in strength. Additionally, physical treatments like multi-step stretching can avoid using additives. In the wet-spinning production, stretching can increase crystallinity by improving molecular orientation in fibers, resulting in higher stiffness and tensile strength [21,22,23,24]. An ultra-stiff chitosan fiber is reported with a modulus as high as 44.7 GPa [18].

Since more than 43.1% of alginate products are designed for pharmaceutical applications, strict evaluation is required for the additives concerning their stability, effectiveness, neurotoxicity, immunogenicity, and biosafety in the short and long terms [25]. For in vivo implants, undegradable additives will retain in local tissue sites after alginate degradation. Decreased biodegradability will cause concerns for the biosafety of reinforced materials.

**Scheme 1:**
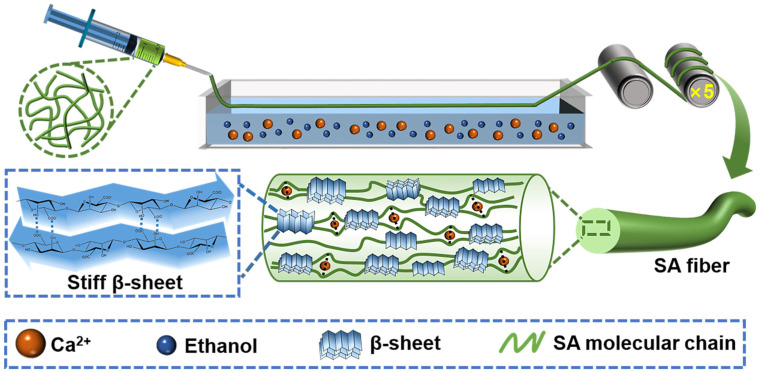
A schematic diagram of wet-spinning process to fabricate self-reinforced alginate fibers. During fibrous formation, the secondary structure of SA was regulated into stiff β-sheet.

This work is designed to address the two major problems in the reinforcement of alginate fibers, namely (1) an effective method to reinforce alginate fibers, as well as (2) preserving the inherent degradability of alginate. In nature, protein-based fibers always simultaneously exhibit robust tensile strength and higher modulus due to a high content of β-sheet in secondary structures [26,27]. β-sheet structures in proteins are usually stiff with high strength [28,29,30]. Under inspiration, this work focuses on a self-reinforcing strategy, which solely involves ethanol-regulated secondary structures of SA. By adapting a coagulation bath composed of CaCl_2_ and ethanol, the secondary structure of SA was regulated during the fibrous formation to fabricate self-reinforced alginate fibers (Scheme 1). Ethanol fast retracted water molecules from SA networks and formed stiff β-sheet structures. It improved the modulus and strength of alginate fibers. The self-reinforced alginate fibers exhibited an increment of 39.0% in tensile strength and 71.9% in toughness. The self-reinforcing strategy also preserved the degradability of the alginate. This work provides a fundamental understanding for β-sheet structures in polysaccharides and subsequent self-reinforcing mechanisms. It is significant for synthesizing strong and tough fibers with minimal changes to the wet-spinning process.

## 2. Experimental Section

### 2.1. Materials

Sodium alginate (SA of 66 and 273 kDa) was obtained from Qingdao Hyzlin Biology Development Co., Ltd (Qingdao, China). The mannuronic/guluronic ratio was 1.3. Ethanol (≥99%) and CaCl_2_ (99.9%) were purchased from Sinopharm Group (Beijing, China). Deionized water was used for all solution preparations.

### 2.2. Secondary Structure Regulation of Sodium Alginate by Ethanol

SA solution with a concentration of 10^−3^ g mL^−1^ was prepared and stirred for 2–3 h to ensure even dispersion. The pH of dispersion was around 6.5. For ethanol-regulated secondary structure, the volume ratio of ethanol to SA solution was set at 30, 40, 50, 60, and 70%, respectively. All the samples were gently stirred for circular dichroism (CD) spectral analysis. For rheological analysis, the concentration of SA was 10^−3^ g mL^−1^.

For X-ray diffraction (XRD) characterization, SA solution of 0.5 wt% was used to prepare different samples. For ethanol-regulated SA, the volume ratio of ethanol was 50%. After gently stirring, all of the samples were collected by centrifugation at 3000 r min^−1^ for 10 min and then dried in vacuum.

### 2.3. Preparation of Self-Reinforced Alginate Fibers

The spinning dope was the solution of 4 wt% SA of 273 kDa, in which the polymer chains formed heavily entangled networks. Then the spinning solution was extruded into a coagulation bath through a syringe needle of 0.7 mm in diameter and 2 cm in length. The fibers were instantly formed and collected. The draft ratios of SA fibers were set at 1:1, 2:1, 3:1, 4:1, and 5:1, respectively. Two kinds of coagulation baths were set. For the control group of SA fibers, the coagulation bath only contained 5 wt% of CaCl_2_ solution. For ethanol-regulated group, the coagulation baths were a solution of 5 wt% CaCl_2_ mixed with different volume ratios of ethanol. All fibers were washed with water before collecting to avoid fusion among fibers. The dried fiber samples were subjected to structural characterization and mechanical properties testing. In the mechanical test, each fiber sample was measured 10 times with an automated single fiber system (FAVIMAT-AIROBOT, TEXTECHNO Company, Moenchengladbach, Germany). The linear density and mechanical properties were automatically obtained at strain rate of 20 mm min^−1^. The tensile modulus was calculated by the average slope of stress–strain curves in the strain range of 0.5–1.5%.

### 2.4. Characterizations

Fourier transform infrared spectroscopy (FT-IR, Nicolet iS5, Thermo Scientific Company, Waltham, MA, USA) is recorded on the spectrometer with a wavenumber range of 400–4000 cm^−1^.

The scanning electron microscope (SEM) test was carried out using a field emission scanning electron microscope (Quanta250FEG, FEI-SEM, Hillsboro, OR, USA). The samples were sprayed with gold in a GVC-2000 ion sputtering instrument before measurement.

The water contact angle test was performed using the optical contact angle tester (Theta, Biolin, Sweden). The water was dropped onto the sample through a syringe. The samples were dried films obtained from solutions containing ethanol. Free surface energy by OWRK/Fowkes method was also provided in Table S1, and diiodomethane was used as the second liquid [31]. The test was repeated three times at different positions of the sample and the final results gave the corresponding average value.

For circular dichroism (CD, J-1500, JASCO Corporation, Tokyo, Japan), the concentration of samples was set at 10^−3^ g mL^−1^ to ensure transparency during the measurement. All the CD spectra were automatically subtracted by corresponding blank samples. The blank samples contained the same chemicals of corresponding SA samples except with no SA inside. SA solution samples were tested in a nitrogen atmosphere with CD in the range of 180–250 nm.

Gel permeation chromatography (GPC, TDA 305, Malvern, Germany) tests were performed using 0.1 M NaNO_3_ as the mobile phase, and the concentration of SA was set at 1 mg mL^−1^.

The X-ray diffractometry (XRD) test of the SA samples was characterized using an X-ray diffractometer (Smart Lab 3KW, Rigaku Corporation, Osaka, Japan). The X-ray source is Cu Kα1 (λ = 1.5406 Å). Test conditions were as follows: the voltage was 60 kV, the electric current was 60 mA, the scanning speed was 5° min^−1^ for 2θ = 5°–40°.

Rheology (DHR-3, TA, New Castle, DE, USA) measurements were performed using a 60 mm sample stage with a sample thickness of 350 μm. For the viscosity test, the shear rate range at 25 °C was 0.1–1000 s^−1^. The storage modulus (*G*′) and loss modulus (*G*″) based on dynamic frequency were tested at angular frequencies (ω) of 0.1–628 rad s^−1^.

The tensile test of SA fiber samples prepared under different conditions was carried out by automated single fiber system. According to the test curve, the stress and strain at the fracture of the sample were taken as the tensile strength and elongation at break. The area under the test curve was the toughness of the fiber. A continuous tensile loading-unloading cycle experiment was carried out at a constant rate of 5 mm min^−1^.

The creep deformation of fibers was tested by dynamic mechanical analysis (DMA, DMA850, TA, New Castle, DE, USA) at constant tensile stress of 30, 40 cN, respectively.

## 3. Results and Discussion

### 3.1. Constructing β-Sheet in SA Using Ethanol Treatment

Secondary structures could be regulated in the molecular assembly of SA of low molecular weight (*M_w_*) to acquire nano-/micro-sized aggregates, which can then be further embedded into alginate fibers as reinforcing units [32]. Unlike this, in the wet-spinning process, SA of high *M_w_* requires effective methods to synchronously regulate secondary structures in fibrous formation. When SA of 273 kDa was treated with ethanol (EtOH), the featuring peaks of C=O and C-O stretching vibration at 1590 and 1024 cm^−1^ was unchanged (Figures S1 and S2). As a poor solvent for SA, ethanol could quickly extract water molecules from SA and increase the water contact angle from 43.3 to 48.4° (Figure S3, Table S1) [31,33].

**Figure 1:**
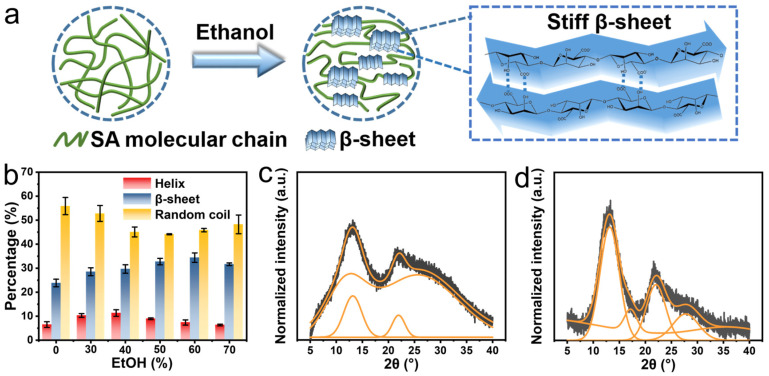
Regulation of SA secondary structure by ethanol. (**a**) A cartoon diagram of SA conformation change post treatment with ethanol. (**b**) The content of secondary structure for ethanol-treated SA at different volumetric ratios. XRD pattern of (**c**) SA and (**d**) SA treated by ethanol. The yellow lines represented fitted curves of XRD pattern.

Due to the changed symmetric distribution of carboxyl chromophore in different conformations, the n→π* interaction generated characteristic CD signals at 192 and 208–215 nm, making the content of β-sheet structures estimable (Figure S4, Table S2) [34,35,36,37]. As evaluated by the CD spectrum, SA without treatment possessed a relatively high content of random coil, an ultra-low proportion of helix, and hybrid β-sheet structures (right-twisted antiparallel β-sheet, parallel β-sheet, and relaxed ones) (Figure 1a,b, Table S2). When ethanol was added in different volume ratios, the CD signal intensity changed and the negative band position shifted from 210 to 217 nm when ethanol reached 70% (Figure S4a). It has been reported that the negative band at 217 nm is one of the feature signs of a high content β-sheet. The CD spectra of ethanol-treated SA were similar to those of IgG at 80 °C, and some of the literature attributes the deformed shape to the mixture of α-helix and β-sheet [38]. For alginate, the fitted results also indicated the hybrid combination of distorted helix and β-sheet (Table S2). The content of β-sheet was increased from 23.8 ± 1.1 to 34.4 ± 2.0%, when the volume of ethanol was increased from 0 to 60% (Figure 1b and Table S2). Random coil was gradually consumed during ethanol addition. The content of helix did not change significantly in the range of 6.5–11.3%. But when the volumetric ratio of ethanol reached 70%, a decreased content of β-sheet was observed, which may relate to the poor dispersion of SA in high concentrations of ethanol.

Ethanol-treated SA samples were further tested using X-ray diffraction (XRD) (Figure 1c,d). Before being treated with ethanol, SA mainly possessed two wide peaks at 2θ = 13.2 and 22.1°. After ethanol treatment, the major peaks became narrower and shifted to 2θ = 13.2, 22.1, and 27.7° (Figure 1d). The full width at half maximum (FWHM) at 2θ = 13.2° was decreased from 4.3 ± 0.3° to 3.1 ± 0.5° (Figure S5).

### 3.2. Rheological Measurement for Self-Reinforcing Mechanism

**Figure 2:**
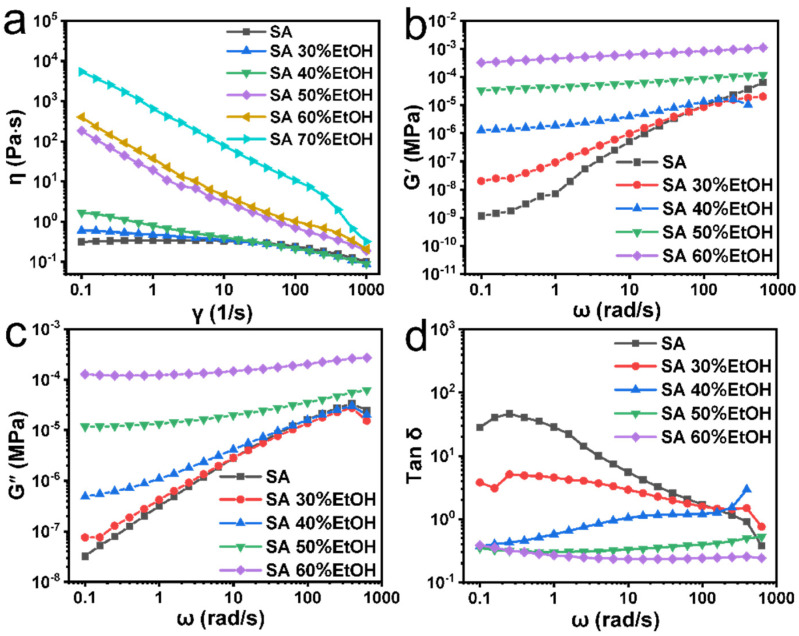
Rheological measurement of ethanol−treated SA dispersion. (**a**) Viscosity (*η*), (**b**) storage modulus (*G*′), (**c**) loss modulus (*G*″), and (**d**) loss tangent (tan *δ*) of SA treated with ethanol at different volumetric ratios. The molecular weight of SA was 273 kDa. The concentration of SA was 10.0 mg mL^−1^.

The reinforcing mechanism for secondary structures was unclear in polysaccharides. Therefore, the influence of β-sheet on the dissipation networks was also studied using rheological tests (Figure 2). When the SA dispersion was treated with an increasing volumetric ratio of ethanol from 30 to 60%, the shear-thinning effects of non-Newtonian fluid were observed for all samples (Figure 2a) [39]. At a low shearing frequency, the flat area of apparent viscosity (*η*) disappeared with increased concentrations of ethanol, which was around 0.3 Pa·s for SA without treatment. When the ethanol reached 50% (*v/v*), thixotropic gelation behavior was obviously observed as *η* continuously decreased with increased frequency. The viscoelasticity for the SA samples was also studied through dynamic rheological analysis. An angular frequency-dependent increase in storage modulus (*G*’) and loss modulus (*G*″) was observed for SA treated with different concentrations of ethanol (Figure 2b–d). Both *G*′ and *G*″ in low frequency exhibited stepwise increases with increased concentrations of ethanol. This confirmed that ethanol could produce stiff β-sheet, whose modulus was much higher than untreated SA. In addition, the phase angle (tan *δ* = *G*″/*G*′) could also reveal some structural information (Figure 2d). *G*′ was constantly larger than *G*″, which resulted into tan *δ* < 1, confirming the gelation of SA [40]. These curves could not overlap when ethanol was higher than 50% (*v/v*), indicating the difficulty of destroying stiff β-sheet in the networks. The disappearance of internal friction peaks in tan *δ* curves also confirmed the inability of motion or destruction at the current angular frequency range of 0.1–628 rad s^−1^ (Figure 2d). Hence, ethanol treatment increased stiff β-sheet structure to enhance the modulus and mechanical strength for SA networks.

### 3.3. Fabrication and Application of Self-Reinforced Alginate Fibers

**Figure 3:**
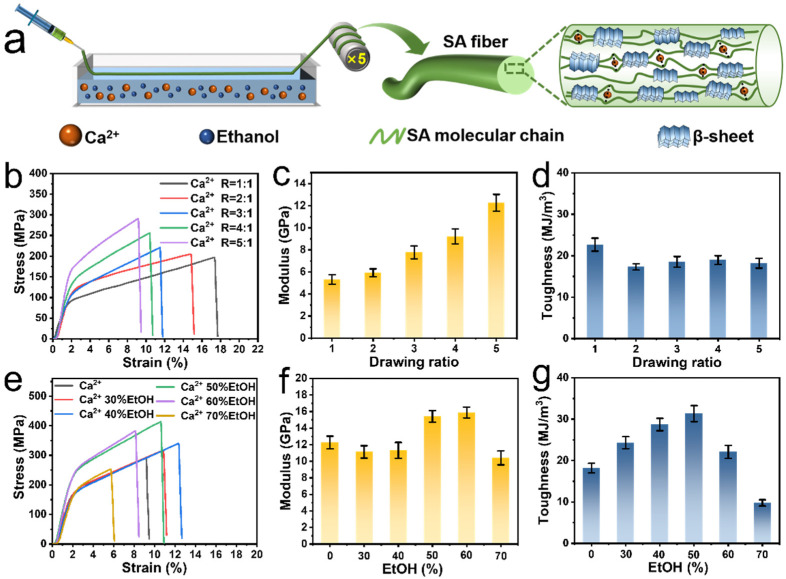
(**a**) Schematic illustrations of wet-spinning process to produce self-reinforced alginate fibers. The mechanical property of fibers fabricated from different coagulation baths, including stress–strain curves (**b**,**e**), calculated modulus (**c**,**f**) and toughness (**d**,**g**) based on stress–strain curves. The compositions of the coagulation bath were 5 wt% of CaCl_2_ (**b**–**d**) and 5 wt% of CaCl_2_ mixed with different volumetric ethanol (**e**–**g**). The spinning dope was 4 wt% of 273 kDa SA. The draw ratio was set at 5:1 for the fibers obtained in mixed coagulation bath.

In a typical wet-spinning process, the spinning solution containing 4 wt% of SA was extruded into a coagulation bath of 5 wt% CaCl_2_ (Figure 3a). Carboxylate groups were quickly cross-linked by Ca^2+^ and SA became solid fibers instantly (Figure S6). The drawing ratio was an important parameter to increase tensile strength by optimizing molecular orientation. When the drawing ratio was increased from 1 to 5, the tensile strength of the SA fibers was increased from 192.2 ± 14.1 to 295.1 ± 16.7 MPa, and the modulus was increased from 5.3 ± 0.4 to 12.3 ± 0.8 GPa, respectively (Figure 3b,c, Table S3). But the elongation decreased from 16.7 ± 1.0 to 9.7 ± 1.6% and corresponding toughness also decreased from 22.7 ± 1.6 to 18.2 ± 1.2 MJ m^−3^ (Figure 3d). Considering the relatively high strength, the drawing ratio was set at 5 in the following wet-spinning process.

Since stiff β-sheet could be constructed by ethanol, the CaCl_2_ coagulation bath was changed accordingly to achieve secondary structure regulation during fibrous formation. When ethanol was added into the CaCl_2_ coagulation bath, both tensile strength and the elongation of alginate fibers increased with increasing ethanol volumetric ratios to 40%, where the highest elongation reached 12.9 ± 1.1% (Figure 3e, Table S3). With the continuing increase of ethanol, elongation began to decrease, while tensile strength reached its highest value as 410.2 ± 9.5 MPa at 50% of ethanol. When the ethanol ratio was higher than 60%, alginate fibers exhibited decreased mechanical properties in both strength and elongation, which may result from inhibited even structural formation due to the fast water extraction from the system [41]. But the modulus reached highest values at 15.4 ± 0.7 and 15.9 ± 0.7 GPa for fibers from 50 and 60% of ethanol (Figure 3f). The toughness also reached a top value of 31.3 ± 1.9 MJ m^−3^ at 50% of ethanol (Figure 3g). The self-reinforced alginate fibers exhibited increments of 39.0% in tensile strength and 71.9% in toughness at 50% of ethanol. The best volumetric ratio of ethanol was set at 50% in the following study. Hence, stiff β-sheet introduced by ethanol can promote the modulus of alginate fibers, thus enhancing the tensile strength and toughness.

**Figure 4:**
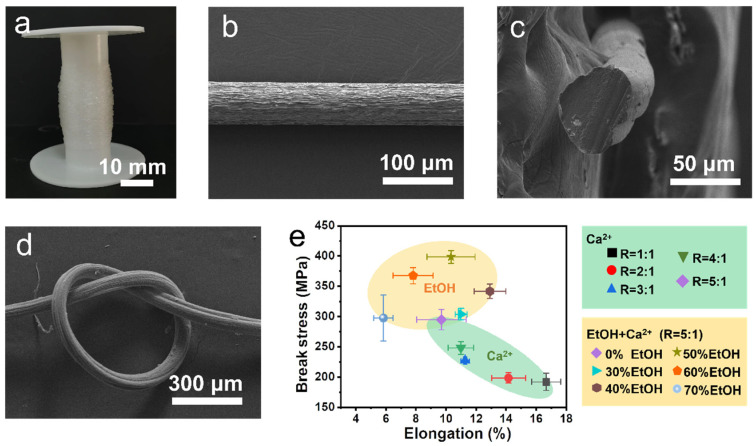
Morphological observation of self-reinforced alginate fibers and comparison of the mechanical properties. (**a**) An optical image of collected self-reinforced alginate fibers. SEM photos for the surface (**b**), and cross-section view (**c**) of self-reinforced alginate fibers. (**d**) An SEM image of knotted self-reinforced alginate fibers. (**e**) A diagram of mechanical property distribution of alginate fibers fabricated with different parameters.

The self-reinforced alginate fibers could be produced in high quantity due to only slight changes in the coagulation bath (Figure 4a). The scanning electron microscope (SEM) photos showed good fibrous morphology with some grooves on the surface (Figure 4b), which may be caused by the chain shrinkage in crosslinking [42]. The fiber was, on average, 59.3 ± 7.4 μm in diameter. The cross-section view was dense and no noticeable defects, core-sheath dislocation, or phase separation was observed (Figure 4c). The self-reinforced alginate fibers maintained good flexibility for knotting (Figure 4d).

The relationship between mechanical properties and secondary structure was compared comprehensively for self-reinforced alginate fibers (Table 1). A critical feature was the greatly improved breaking tensile stress (Figure 4e). The strengthening effect was obvious for alginate fibers treated with ethanol, where the high content of stiff β-sheet (32.8%) was the main contributor (Figures S7 and S8). The mechanisms of β-sheet-influenced mechanical properties were similar to those of protein-based fibers. Table 1 summarizes the contents of helix, β-sheet, and random coil for a few spider silk fibers, regenerated silk fibroins fibers, silkworm silk fibers, and regenerated wool keratin fibers [28,30,43,44,45,46,47,48]. Apart from the different reinforcing mechanisms reported in the literature, the changes in mechanical properties presented similar positive correlations for tensile strength versus β-sheet contents. For example, spider silk fibers of SUMO-NT-R-CT contained higher contents of β-sheet (35.6%) than fibers of SUMO-NT_A72S_-R-CT_C92S_ (29%), and the tensile strength was enhanced from 103 to 1007 MPa [44]. In addition, regenerated silk fibroins fibers of RSF-SN contained higher contents of β-sheet (55.8%) than fibers of *B. mori* silk (44.2%), and the tensile strength was enhanced from 610 to 2054 MPa [30]. Similar positive correlations could also be analyzed from other protein-based fibers in Table 1. These results were in good consistence with self-reinforcing mechanisms of alginate fibers in this work, successfully expanding the applicable scope of secondary structures from proteins to polysaccharides.

The mechanical properties were further compared with other reinforced fibers based on polysaccharides and proteins (Table 2). For self-reinforced alginate fibers, the increment of tensile strength was 39.0%, which was higher than most fibers, including hydroxyapatite/alginate fibers, sodium polyacrylate/alginate fibers, kapok/alginate fibers, hemp/alginate fibers, nanofibrillated chitin (NFCh) macrofiber, Ag Nanowires/silkworm silk fibers, cellulose nanofibers (CNF)/recombinant spider silk proteins (RSP), bacteria cellulose nanofibers/alginate fibers, carrageenan fibers, and other reinforced fibers [10,11,15,20,49,50,51,52,53]. But the increment was significantly lower than with oxidized SA/calcium alginate/Antarctic krill protein composite fibers (44.4%), graphene oxide (GO)/alginate fibers (93.8%), nanofibril engineered chitin fibers (68.9%), or cellulose nanofibers (CNF) (69.5%) [54,55,56,57]. Although the strength increment of some other fibers was higher than that of the self-reinforced alginate fibers, the elongation showed a negative growth (Table 2). This result confirmed that the self-reinforcing strategy could efficiently enhance mechanical properties by embedding stiff β-sheet.

**Figure 5:**
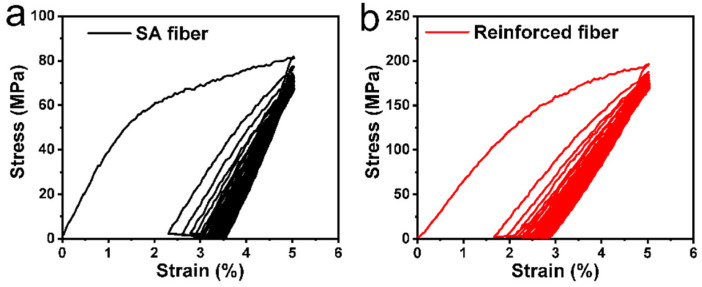
The mechanical property of cyclic strain of 5% for 30 times. The corresponding stress–strain curves for SA fibers (**a**) and self-reinforced alginate fibers (**b**). The drawing speed was 5 mm min^−1^.

The elastic resilience was also improved for self-reinforced alginate fibers. Under the repeated drawing of 5% of strain, the hysteresis area was 15.7% less than that of SA fibers in the first cycle (Figure 5). After 30 cycles, the tensile strength was only reduced by 14.3%, and it was 18.2% for SA fibers. The crept percentage was only 2.8%, while it was 3.6% for that of SA fibers. Under constant tensile stress of 30 and 40 cN, self-reinforced fibers could achieve shorter elongation and time duration to equilibrate than SA fibers in dynamic mechanical analysis (DMA) curves (Figure S9).

**Figure 6:**
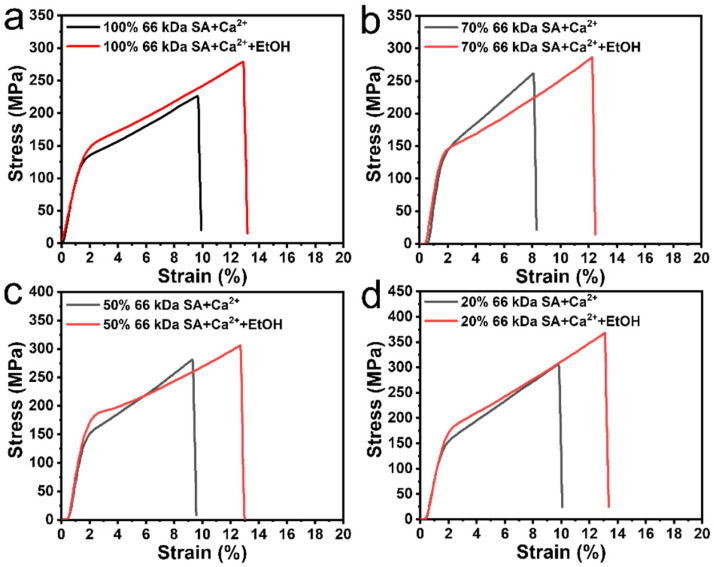
The stress–strain curves of mixed SA of 273 kDa and 66 kDa at different ratios. The ratio of 66 kDa SA to total SA was 20% (**a**), 50% (**b**), 70% (**c**), and 100% (**d**). The total SA concentration of the spinning dope was set at 4 wt%.

In addition, the mechanical properties of low *M_w_* SA fibers were generally lower than those of high *M_w_* SA fibers. The self-reinforced strategy can also produce self-reinforced fibers from high and low *M_w_* mixed SA, as well as pure low *M_w_* SA (Figure 6). When 20, 50, 70, and 100% of 273 kDa SA were replaced by SA of 66 kDa, the tensile strength was improved by 22.8, 7.2, 14.5, and 23.0%, respectively (Table S4). Their elongation remained high in the range of 12.2–13.1%. It should be noted that the tensile strength of the SA fibers of 273 kDa was 295.1 ± 16.7 MPa. Furthermore, 50% of high *M_w_* SA could be replaced with low *M_w_* SA, without causing a decrease in mechanical properties. Hence, a self-reinforcing strategy may decrease the industrial cost by using hybrid SA of high and low *M_w_* (Table S5). Though not explored, the fracture mechanics of reinforced alginate fibers was also important to fully understand the mechanical behavior of β-sheet. Some strong methods, such as the Bezier multi-step method and differential quadrature method, may provide numerical evaluation concerning the reinforced composite structures based on the reported data [60,61].

Another advantage of the self-reinforcing strategy is the well-preserved degradability of the reinforced alginate fibers. The calcium ions cross-linked alginate can slowly dissolve into physical media due to calcium ions exchanged by monovalent ions. After being incubated in saline after storage for 7 days, self-reinforced alginate fibers transformed from a solid state into a dissolved gel-like dispersion (Figure S10). This evidence confirmed the well-preserved degradability of self-reinforced alginate fibers.

## 4. Conclusions

In summary, a self-reinforcing strategy has been proven effective to enhance the mechanical properties and preserve the degradability of alginate fibers. There are many important parts involved in this paper. (1) Ethanol can construct β-sheet conformation for SA. (2) β-sheet is stiff and can improve the modulus of SA physical networks. (3) By using a coagulation bath of ethanol and CaCl_2_, stiff β-sheet can effectively enhance the tensile strength and toughness of alginate fibers. (4) The self-reinforced alginate fibers preserved the degradability of alginate, exhibiting promising futures in biological applications. This work provides fundamental understanding for β-sheet structures in polysaccharides and subsequent self-reinforcing mechanisms. In the future, great efforts are still needed to develop new methods to construct other secondary structures in polysaccharides for enhanced mechanical properties. The in vivo bioactivity, degradation, and biosafety is also worth exploring for reinforced alginate fibers.

## Figures and Tables

**Table 1:** A list of secondary structure contents and mechanical properties for some representative protein-based fibers.

Fiber type		β-Sheet (%)	*Random coil* (%)	Helix (%)	Stress (MPa)	Modulus (GPa)	Toughness (MJ m^−3^)	Ref.
SA fibers	SA fibers	23.8 ± 1.6	55.9 ± 3.6	6.5 ± 1.2	295.1 ± 16.7	12.3 ± 0.8	18.2 ± 1.2	This work
	50% EtOH	32.8 ± 0.4	44.2 ± 0.2	8.9 ± 0.4	410.2 ± 9.5	15.4 ± 0.7	31.3 ± 1.9	
Spider silk fibers	SUMO-NT_A72S_-R-CT_C92S_	29	17	17.8	103.0 ± 10.0	4.0 ± 0.9	10.0 ± 1.0	[44]
	SUMO-NT-R-CT	35.6	17.5	19.2	1007 ± 77	36.7 ± 9.0	191 ± 26	
	A_15_-A_14_	40.1	40.2		44.1 ± 19.6	1.7 ± 0.6	18.2 ± 20.3	[43]
	(A_3_I)_3_-A_14_	43.2	30.3		131.6 ± 31.9	3.5 ± 0.9	145.6 ± 42.2	
Regenerated silk fibroins fibers	Bending angle 0°	25.9 ± 1.7	62.7 ± 1.4		1205 ± 78	11.1 ± 1.6	130.9 ± 24.2	[45]
	Bending angle 90°	33.2 ± 0.9	56.5 ± 2.1		1304 ± 178	13.4 ± 1.5	122.3 ± 38.6	
	RSF-SN	55.8 ± 1.6	14.8 ± 2.2	14.6 ± 1.0	2054 ± 177	43 ± 6	155.0 ± 34	[30]
	RSF-Pa	51.3 ± 1.1	17.3 ± 0.8	17.3 ± 0.3	816 ± 124	21 ± 3	215.8 ± 29	
	*B. mori* silk	44.2 ± 1.1	22.1 ± 0.6	16.1 ± 0.3	610 ± 84	16 ± 4	55.3 ± 12.4	
Silkworm Silk fibers	*B. mori* silk	45 ± 2.5	38 ± 3.5		560 ± 80	11.0 ± 1.7	69 ± 25	[28]
	Hot-stretched silkFibers (7.5%-S)	49 ± 5	32.5 ± 7.5		770 ± 130	21.6 ± 2.8	80 ± 46	
Regenerated wool keratin fiber	Dry conditions	65.4 ^a^	--	34.6 ^a^	22.6 ± 2.7	--	--	[47]
	Wet conditions	34.5 ^a^	--	65.5 ^a^	2.71 ± 0.3	--	--	
	5 wt% PEGDA and 40 wt% cortical cells	60.5		39.5	8.3 ± 1.0 cN/tex	--	--	[46]
	GA/MDI crosslinked regenerated fibers	63.3		36.7	10.0 ± 1.2 cN/tex	--	--	
	Drawing ratio 2.4	17.8 ^a^	--	82.2 ^a^	144.9 ± 4.9	5	13.4	[48]
	Drawing ratio 4.8	39.4 ^a^	--	60.6 ^a^	186.1 ± 7.1	7.4	10.9	

^a^ refers to specific ratio of helix and β-sheet.

**Table 2:** A comparison of mechanical properties for self-reinforced alginate fibers and other reinforced biomass fibers.

Fiber Type	Additives	Control Group			Reinforced Fibers				
		Elongation (%)	Strength (MPa)	Toughness (MJ m^−3^)	Elongation (%)	Strength (MPa)	Toughness (MJ m^−3^)	Strength Increment (%)	Ref.
This work	--	9.7	295.1	18.2	11.2	410.2	31.3	39.0	This work
Alginate fibers	Hydroxyapatite	23.5	121.4	15.7	20.6	153.8	18.5	26.7	[10]
	Sodium polyacrylate	11.8	0.57 cN/dtex	5.6	7.0	0.64 cN/dtex	3.5	12.3	[15]
	Antarctic krill protein	12.0	1.8 cN/dtex	21.9	9.5	2.6 cN/dtex	26.8	44.4	[54]
	Kapok fibers	2.1	135 cN	--	1.2	174 cN	--	28.9	[49]
	Hemp fibers	2.1	135 cN	--	1.5	185 cN	--	37.0	
	Graphene oxide fibers	16.8	320	--	14.3	620	--	93.8	[55]
	Graphene fibers	7.6	199	--	5.6	203.8	--	2.4	[58]
	Alginate fibers	16.0	130	22.0	18.0	173	23.0	33.1	[59]
	Bacterial cellulose	7.9	394.2	16.0	12.2	535.4	38.3	35.8	[20]
		7.9	394.2	16.0	13.0	504.2	37.1	27.9	
Chitin nanofibril fibers	--	9.5	132.0	--	3.5	223	--	68.9	[56]
Chitin nanofibril fibers	--	8.6	1.7 cN/dtex	--	5.2	2.3 cN/dtex	--	35.3	[50]
Cellulose nanofibers	--	5.8	131	--	2.9	222	--	69.5	[57]
Bacterial cellulose	--	4.5	198	--	3.8	248.6	--	25.6	[52]
Cellulose nanofibers	Spider silk proteins	6.1	830	43.0	10.5	1015	55.0	22.3	[51]
Silkworm silk	Ag Nanowires	17.8	430	58.1	24.5	590	98.1	37.2	[11]
Silkworm silk	--	16.4	560	69.0	11.9	770	80.0	37.5	[28]
Carrageenan fibers	--	6.6	0.75 cN/dtex	--	9.6	0.73 cN/dtex	--	−2.7	[53]

## Data Availability

The original contributions presented in the study are included in the article/Supplementary Materials, further inquiries can be directed to the corresponding author.

## Supplementary Materials

The following supporting information can be downloaded at: https://www.mdpi.com/article/10.3390/ma17133047/s1, Figure S1. GPC revealed molecular weight for SA of 66 and 273 kDa. Figure S2. FT-IR spectra SA and ethanol treated SA. Figure S3. Water contact angle of SA before and after ethanol treatment. Figure S4. CD spectrum (a) and absorption spectra (b) of ethanol treated SA at different volumetric ratios. The molecular weight of SA was 273 kDa. The concentration of SA was 1.0 mg mL^−1^. Figure S5. The full width at half maximum (FWHM) of fitted peak at 2θ = 13.2° and 22.1° for samples of SA and ethanol treated SA. Figure S6. SEM photo of SA fibers fabricated from coagultion bath of 5 wt% of CaCl_2_. Figure S7. The fitted curve of the β-sheet content (*C_β_*) in SA versus enhanced percentages of fiber tensile strength (*P_s_*) after treated by ethanol. Figure S8. The fitted curve of the β-sheet content (*C_β_*) in SA versus enhanced percentages of fiber toughness (*P_t_*) after treated by ethanol. Figure S9. DMA measurement for time dependent elongation of SA fibers and self-reinforced fibers at constant tensile stress of 30 cN (a) and 40 cN (b). Figure S10. Optical images of self-reinforced alginate fibers soaked in saline before (a) and after (b) 7 days of storage. The concentration of fibers was 20 mg/mL. Before soaking in saline, the pH of wet fiber was ~6. 7 days later, the pH of saline was changed to 6.1. Table S1. Water contact angle test for different SA samples. Table S2. A list for fitted content of secondary structures ^a^. Table S3. A list of mechanical properties of SA fibers and reinforced alginate fibers. Table S4. The mechanical property of SA fibers and reinforced alginate fibers when different proportions of high *M_w_* SA was replaced by low *M_w_* SA. Table S5. The *M_w_* and yield of SA extracted from brown seaweeds from different locations. Refs. [62,63,64,65,66] are cited in the supplementary materials.

## References

[1] Koutsopoulos S. (2012). Molecular fabrications of smart nanobiomaterials and applications in personalized medicine. Adv. Drug Deliv. Rev..

[2] Guan W., Gong C., Wu S., Cui Z., Zheng Y., Li Z., Zhu S., Liu X. (2023). Instant protection spray for anti-Infection and accelerated healing of empyrosis. Adv. Mater..

[3] Huang C., Xie T., Liu Y., Yan S., Ou Yang F., Zhang H., Lei L., He D., Wei H., Yu C.Y. (2023). A sodium alginate-based multifunctional nanoplatform for synergistic chemo-immunotherapy of hepatocellular carcinoma. Adv. Mater..

[4] Lei X.-X., Hu J.-J., Zou C.-Y., Jiang Y.-L., Zhao L.-M., Zhang X.-Z., Li Y.-X., Peng A.-N., Song Y.-T., Huang L.-P. (2023). Multifunctional two-component in-situ hydrogel for esophageal submucosal dissection for mucosa uplift, postoperative wound closure and rapid healing. Bioact. Mater..

[5] Zhang M., Qiao X., Han W., Jiang T., Liu F., Zhao X. (2021). Alginate-chitosan oligosaccharide-ZnO composite hydrogel for accelerating wound healing. Carbohydr. Polym..

[6] Yi Y., Song J., Zhou P., Shu Y., Liang P., Liang H., Liu Y., Yuan X., Shan X., Wu X. (2024). An ultrasound-triggered injectable sodium alginate scaffold loaded with electrospun microspheres for on-demand drug delivery to accelerate bone defect regeneration. Carbohydr. Polym..

[7] Lim J., Choi G., Joo K.I., Cha H.J., Kim J. (2021). Embolization of vascular malformations via In situ photocrosslinking of mechanically reinforced alginate microfibers using an optical-fiber-integrated microfluidic device. Adv. Mater..

[8] Tian X., Zhu H., Meng X., Wang J., Zheng C., Xia Y., Xiong Z. (2020). Amphiphilic calcium alginate carbon aerogels: Broad-spectrum adsorbents for ionic and solvent dyes with multiple functions for decolorized oil–water separation. ACS Sustain. Chem. Eng..

[9] Zhao X., Ding M., Xu C., Zhang X., Liu S., Lin X., Wang L., Xia Y. (2021). A self-reinforcing strategy enables the intimate interface for anisotropic alginate composite hydrogels. Carbohydr. Polym..

[10] Wan F., Ping H., Wang W., Zou Z., Xie H., Su B.-L., Liu D., Fu Z. (2021). Hydroxyapatite-reinforced alginate fibers with bioinspired dually aligned architectures. Carbohydr. Polym..

[11] Lu H., Jian M., Yin Z., Xia K., Shi S., Zhang M., Wang H., Liang X., Ma W., Zhang X. (2022). Silkworm silk fibers with multiple reinforced properties obtained through feeding Ag nanowires. Adv. Fiber Mater..

[12] Wang Z., Zhang Y., Tang P., Deng Z., He P., Chen M.-J., Yu Z.-Z., Zhang H.-B. (2023). Silk fibroin reinforced graphene fibers with outstanding electrical conductivity and mechanical strength. Carbon.

[13] Gao Z., Zhu J., Rajabpour S., Joshi K., Kowalik M., Croom B., Schwab Y., Zhang L., Bumgardner C., Brown K.R. (2020). Graphene reinforced carbon fibers. Sci. Adv..

[14] Wang Q., Wang C., Zhang M., Jian M., Zhang Y. (2016). Feeding single-walled carbon nanotubes or graphene to silkworms for reinforced silk fibers. Nano Lett..

[15] Li S., Chandra Biswas M., Ford E. (2022). Dual roles of sodium polyacrylate in alginate fiber wet-spinning: Modify the solution rheology and strengthen the fiber. Carbohydr. Polym..

[16] Kim H.J., Lee W.J. (2023). The Influence of Cellulose Nanocrystal Characteristics on Regenerative Silk Composite Fiber Properties. Materials.

[17] Zhou W., Zhang H., Liu Y., Zou X., Shi J., Zhao Y., Ye Y., Yu Y., Guo J. (2019). Preparation of calcium alginate/polyethylene glycol acrylate double network fiber with excellent properties by dynamic molding method. Carbohydr. Polym..

[18] Chen Y., Zhang Q., Zhong Y., Wei P., Yu X., Huang J., Cai J. (2021). Super-strong and super-stiff chitosan filaments with highly ordered hierarchical structure. Adv. Funct. Mater..

[19] Boroujeni F.M., Fioravanti G., Kander R. (2024). Synthesis and Characterization of Cellulose Microfibril-Reinforced Polyvinyl Alcohol Biodegradable Composites. Materials.

[20] Gao H.-L., Zhao R., Cui C., Zhu Y.-B., Chen S.-M., Pan Z., Meng Y.-F., Wen S.-M., Liu C., Wu H.-A. (2020). Bioinspired hierarchical helical nanocomposite macrofibers based on bacterial cellulose nanofibers. Natl. Sci. Rev..

[21] Li D.-L., Shi S.-C., Lan K.-Y., Liu C.-Y., Li Y., Xu L., Lei J., Zhong G.-J., Huang H.-D., Li Z.-M. (2023). Enhanced dielectric properties of all-cellulose composite film via modulating hydroxymethyl conformation and hydrogen bonding network. ACS Macro Lett..

[22] Li S., Fan Z., Wu G., Shao Y., Xia Z., Wei C., Shen F., Tong X., Yu J., Chen K. (2021). Assembly of nanofluidic MXene fibers with enhanced ionic transport and capacitive charge storage by flake orientation. ACS Nano.

[23] Sun J., Guo W., Mei G., Wang S., Wen K., Wang M., Feng D., Qian D., Zhu M., Zhou X. (2023). Artificial spider silk with buckled sheath by nano-pulley combing. Adv. Mater..

[24] Wen X., Xiong J., Sun Z., Wang L., Yu J., Qin X. (2022). A general strategy to electrospin nanofibers with ultrahigh molecular chain orientation. Engineering.

[25] Fang G., Lu H., Rodriguez de la Fuente L., Law A.M.K., Lin G., Jin D., Gallego-Ortega D. (2021). Mammary tumor organoid culture in non-adhesive alginate for luminal mechanics and high-throughput drug screening. Adv. Sci..

[26] Tan G., Jia T., Qi Z., Lu S. (2024). Regenerated Fiber’s Ideal Target: Comparable to Natural Fiber. Materials.

[27] Qiu W., Patil A., Hu F., Liu X.Y. (2019). Hierarchical Structure of Silk Materials Versus Mechanical Performance and Mesoscopic Engineering Principles. Small.

[28] Lu H., Xia K., Jian M., Liang X., Yin Z., Zhang M., Wang H., Wang H., Li S., Zhang Y. (2022). Mechanically reinforced silkworm silk fiber by hot stretching. Research.

[29] Gu L., Jiang Y., Hu J. (2019). Scalable spider-silk-like supertough fibers using a pseudoprotein polymer. Adv. Mater..

[30] Wang J., Fan T., Li X., Hu X., Huang W., Yuan W., Lin Z. (2022). Artificial superstrong silkworm silk surpasses natural spider silks. Matter.

[31] Ismail M.F., Islam M.A., Khorshidi B., Tehrani-Bagha A., Sadrzadeh M. (2022). Surface characterization of thin-film composite membranes using contact angle technique: Review of quantification strategies and applications. Adv. Colloid Interface Sci..

[32] Liu Y., Hou W., Qi P., Yang J., Xie X., Lin M., Xia Y., Nie Z., Sui K. (2022). Hierarchical nano-helix as a new reinforcing unit for simultaneously ultra-strong and super-tough alginate fibers. Carbohydr. Polym..

[33] Zhou Y., Wu L., Tian Y., Li R., Zhu C., Zhao G., Cheng Y. (2020). A novel low-alkali konjac gel induced by ethanol to modulate sodium release. Food Hydrocoll..

[34] Greenfield N.J. (2007). Using circular dichroism spectra to estimate protein secondary structure. Nat. Protoc..

[35] Newberry R.W., Raines R.T. (2017). The n→π* Interaction. Acc. Chem. Res..

[36] Singh S.K., Mishra K.K., Sharma N., Das A. (2016). Direct spectroscopic evidence for an n→π* interaction. Angew. Chem. Int. Ed..

[37] Zhang H., Li Q., Wang S., Yu X., Wang B., Chen G., Ren L., Li J., Jin M., Yu J. (2022). Confining carbon dots in amino-functionalized mesoporous silica: N→π* interaction triggered deep-red solid-state fluorescence. Nano Res..

[38] Hawe A., Friess W., Sutter M., Jiskoot W. (2008). Online fluorescent dye detection method for the characterization of immunoglobulin G aggregation by size exclusion chromatography and asymmetrical flow field flow fractionation. Anal. Biochem..

[39] Wu Y., Lv B., Wang S., Liu Z., Chen X.D., Cheng Y. (2024). Study of molecular interaction and texture characteristics of hydrocolloid-mixed alginate microspheres: As a shell to encapsulate multiphase oil cores. Carbohydr. Polym..

[40] Su L., Mosquera J., Mabesoone M.F.J., Schoenmakers S.M.C., Muller C., Vleugels M.E.J., Dhiman S., Wijker S., Palmans A.R.A., Meijer E.W. (2022). Dilution-induced gel-sol-gel-sol transitions by competitive supramolecular pathways in water. Science.

[41] Zhang L., Wang S., Wang Z., Liu Z., Xu X., Liu H., Wang D., Tian Z. (2023). Temperature-mediated phase separation enables strong yet reversible mechanical and adhesive hydrogels. ACS Nano.

[42] Qi S., Lin M., Qi P., Shi J., Song G., Fan W., Sui K., Gao C. (2022). Interfacial and build-in electric fields rooting in gradient polyelectrolyte hydrogel boosted heavy metal removal. Chem. Eng. J..

[43] Arndt T., Greco G., Schmuck B., Bunz J., Shilkova O., Francis J., Pugno N.M., Jaudzems K., Barth A., Johansson J. (2022). Engineered spider silk proteins for biomimetic spinning of fibers with toughness equal to dragline silks. Adv. Funct. Mater..

[44] Mi J., Li X., Niu S., Zhou X., Lu Y., Yang Y., Sun Y., Meng Q. (2024). High-strength and ultra-tough supramolecular polyamide spider silk fibers assembled via specific covalent and reversible hydrogen bonds. Acta Biomater..

[45] Peng Z., Hu W., Li X., Zhao P., Xia Q. (2023). Bending–spinning produces silkworm and spider silk with enhanced mechanical properties. Macromolecules.

[46] Cao G.S., Zhang Z.P., Rong M.Z., Zhang M.Q. (2022). A novel strategy for producing high-performance continuous regenerated fibers with wool-like structure. SusMat.

[47] Xu X., Wang Z., Li M., Su Y., Zhang Q., Zhang S., Hu J. (2023). Reconstructed hierarchically structured keratin fibers with shape-memory features based on reversible secondary-structure transformation. Adv. Mater..

[48] Zhu J., Ma N., Li S., Zhang L., Tong X., Shao Y., Shen C., Wen Y., Jian M., Shao Y. (2023). Reinforced wool keratin fibers via dithiol chain re-bonding. Adv. Funct. Mater..

[49] Azam F., Ahmad F., Ahmad S., Zafar M.S., Ulker Z. (2023). Synthesis and characterization of natural fibers reinforced alginate hydrogel fibers loaded with diclofenac sodium for wound dressings. Int. J. Biol. Macromol..

[50] Zhu K., Tu H., Yang P., Qiu C., Zhang D., Lu A., Luo L., Chen F., Liu X., Chen L. (2019). Mechanically strong chitin fibers with nanofibril structure, biocompatibility, and biodegradability. Chem. Mater..

[51] Mittal N., Jansson R., Widhe M., Benselfelt T., Hakansson K.M.O., Lundell F., Hedhammar M., Soderberg L.D. (2017). Ultrastrong and bioactive nanostructured bio-based composites. ACS Nano.

[52] Yao J., Chen S., Chen Y., Wang B., Pei Q., Wang H. (2017). Macrofibers with high mechanical performance based on aligned bacterial cellulose nanofibers. ACS Appl. Mater. Interfaces.

[53] Dong M., Xue Z., Liu J., Yan M., Xia Y., Wang B. (2018). Preparation of carrageenan fibers with extraction of Chondrus via wet spinning process. Carbohydr. Polym..

[54] Li Z., Guo J., Guan F., Yin J., Yang Q., Zhang S., Tian J., Zhang Y., Ding M., Wang W. (2023). Oxidized sodium alginate cross-linked calcium alginate/antarctic krill protein composite fiber for improving strength and water resistance. Colloids Surf. A.

[55] He Y., Zhang N., Gong Q., Qiu H., Wang W., Liu Y., Gao J. (2012). Alginate/graphene oxide fibers with enhanced mechanical strength prepared by wet spinning. Carbohydr. Polym..

[56] Torres-Rendon J.G., Schacher F.H., Ifuku S., Walther A. (2014). Mechanical performance of macrofibers of cellulose and chitin nanofibrils aligned by wet-stretching: A critical comparison. Biomacromolecules.

[57] Hooshmand S., Aitomaki Y., Norberg N., Mathew A.P., Oksman K. (2015). Dry-spun single-filament fibers comprising solely cellulose nanofibers from bioresidue. ACS Appl. Mater. Interfaces.

[58] Fu X., Liang Y., Wu R., Shen J., Chen Z., Chen Y., Wang Y., Xia Y. (2019). Conductive core-sheath calcium alginate/graphene composite fibers with polymeric ionic liquids as an intermediate. Carbohydr. Polym..

[59] Chen Z., Song J., Xia Y., Jiang Y., Murillo L.L., Tsigkou O., Wang T., Li Y. (2021). High strength and strain alginate fibers by a novel wheel spinning technique for knitting stretchable and biocompatible wound-care materials. Mater. Sci. Eng. C.

[60] Bert C.W., Malik M. (1997). Differential quadrature: A powerful new technique for analysis of composite structures. Compos. Struct..

[61] Kabir H., Aghdam M.M. (2021). A generalized 2D Bézier-based solution for stress analysis of notched epoxy resin plates reinforced with graphene nanoplatelets. Thin-Walled Struct..

[62] Fenoradosoa T.A., Ali G., Delattre C., Laroche C., Petit E., Wadouachi A., Michaud P. (2009). Extraction and characterization of an alginate from the brown seaweed Sargassum turbinarioides Grunow. J. Appl. Phycol..

[63] Fertah M., Belfkira A., Dahmane E.M., Taourirte M., Brouillette F. (2017). Extraction and characterization of sodium alginate from Moroccan Laminaria digitata brown seaweed. Arabian J. Chem..

[64] Gomez C.G., Pérez Lambrecht M.V., Lozano J.E., Rinaudo M., Villar M.A. (2009). Influence of the extraction–purification conditions on final properties of alginates obtained from brown algae (*Macrocystis pyrifera*). Int. J. Biol. Macromol..

[65] Torres M.R., Sousa A.P.A., Silva Filho E.A.T., Melo D.F., Feitosa J.P.A., de Paula R.C.M., Lima M.G.S. (2007). Extraction and physicochemical characterization of Sargassum vulgare alginate from Brazil. Carbohydr. Res..

[66] Larsen B., Salem D.M.S.A., Sallam M.A.E., Mishrikey M.M., Beltagy A.I. (2003). Characterization of the alginates from algae harvested at the Egyptian Red Sea coast. Carbohydr. Res..

