# Supplementary material for: Constructing Stiff β-Sheet for Self-Reinforced Alginate Fibers
Source: Materials (Basel). 2024 Jun 21;17(13):3047. doi: 10.3390/ma17133047 (PMC11242387; doi:10.3390/ma17133047)
Supplement: Supplementary file 1 [file materials-17-03047-s001.zip › materials-3058925-supplementary.pdf]

Supporting Information

Constructing stiff β-sheet for self-reinforced alginate fibers

Xuelai Xie [a], Min Cui [a], Tianyuan Wang [a], Jinhong Yang [a], Wenli Li [a], Kai Wang[*,a,b], Min Lin[*,a]

[a] State Key Laboratory of Bio-Fibers and Eco-textiles, College of Materials Science and Engineering, Shandong Collaborative Innovation Center of Marine Biobased Fibers and Ecological Textiles, Qingdao University, Qingdao, 266071, P. R. China.

[b] Institute of Flexible Electronics (IFE), Northwestern Polytechnical University (NPU), Xi'an 710072, P. R. China.

*Corresponding author.

E-mai address: linmin0401@qdu.edu.cn (M. Lin); kaiwang@nwpu.edu.cn (K. Wang)

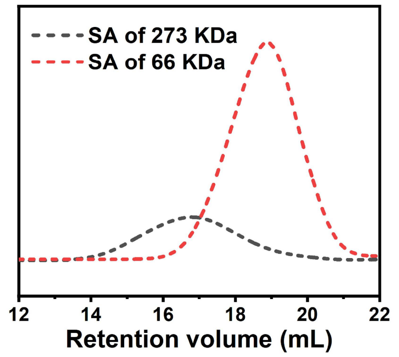

Fig. S1. GPC revealed molecular weight for SA of 66 and 273 kDa.

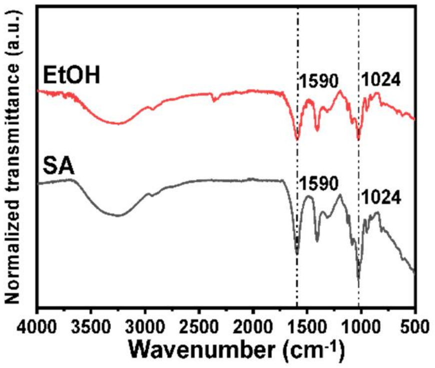

Fig. S2. FT-IR spectra SA and ethanol treated SA.

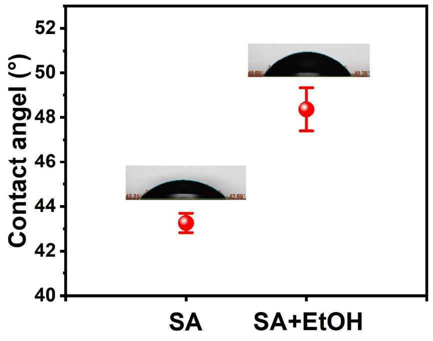

Fig. S3. Water contact angle of SA before and after ethanol treatment.

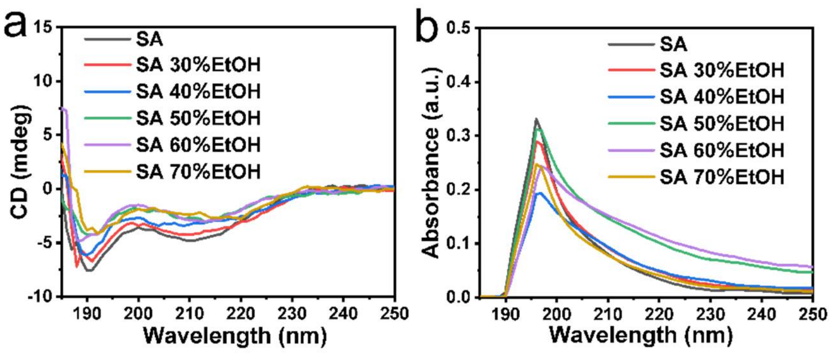

Fig. S4. CD spectrum (a) and absorption spectra (b) of ethanol treated SA at different volumetric ratios. The molecular weight of SA was 273 kDa. The concentration of SA was 1.0 mg mL^{-1}.

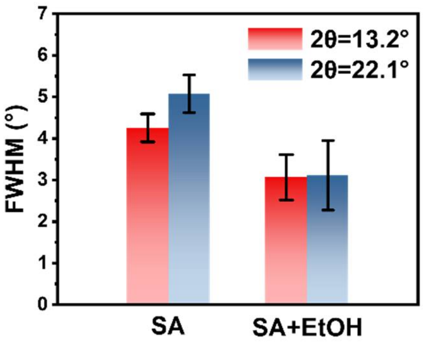

Fig. S5. The full width at half maximum (FWHM) of fitted peak at 2θ=13.2° and 22.1° for samples of SA and ethanol treated SA.

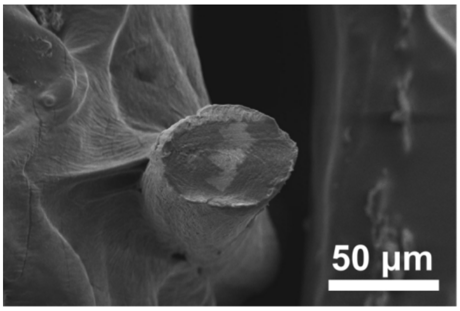

Fig. S6. SEM photo of SA fibers fabricated from coagultion bath of 5 wt% of $CaCl_2$.

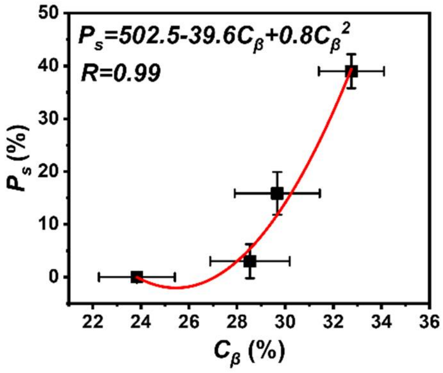

Fig. S7. The fitted curve of the β-sheet content (C_β) in SA versus enhanced percentages of fiber tensile strength (P_s) after treated by ethanol.

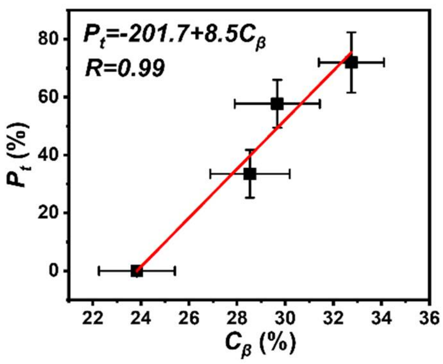

Fig. S8. The fitted curve of the β-sheet content (C_β) in SA versus enhanced percentages of fiber toughness (P_t) after treated by ethanol.

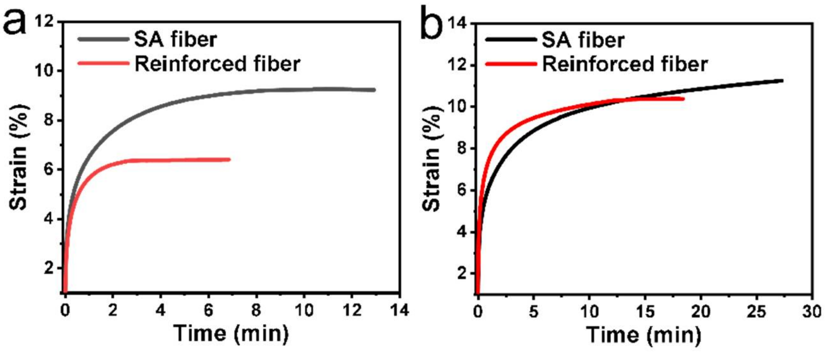

Fig. S9. DMA measurement for time dependent elongation of SA fibers and self-reinforced fibers at constant tensile stress of 30 cN (a) and 40 cN (b).

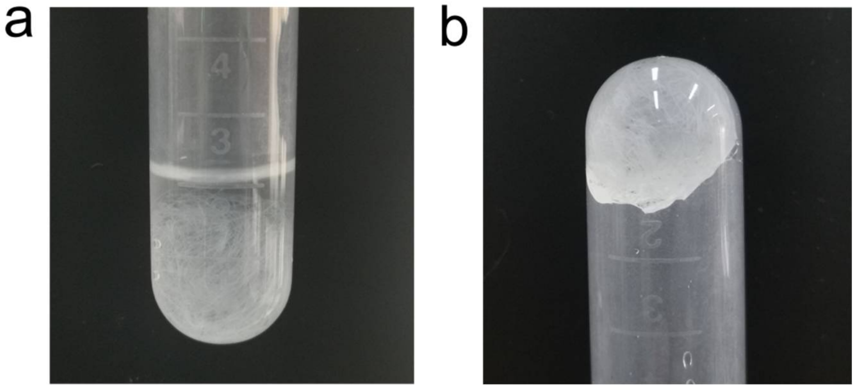

Fig. S10. Optical images of self-reinforced alginate fibers soaked in saline before (a) and after (b) 7 days of storage. The concentration of fibers was 20 mg/mL. Before soaking in saline, the pH of wet fiber was ~6. 7 days later, the pH of saline was changed to 6.1.

Table S1. Water contact angle test for different SA samples.

	Liquid	θ (°)	γ (mN/m)
SA	Water	43.3±0.4	102.2±2.9
SA-EtOH	Water	48.4±0.9	88.0±0.4

Table S2. A list for fitted content of secondary structures[a].

Sample	Helix		Antiparallel			Parallel
	Regular	Distorted	Left-twisted	Relaxed	Right-twisted	
SA	0	6.5±1.3	0	9.1±4.9	12.2±2.3	3.8±0.2
30 % EtOH	0.5±0.9	9.8±0.6	0	0	17.3±0.7	11.2±3.8
40 % EtOH	1.4±1.5	9.9±2.7	0	5.7±2.0	18.3±1.7	8.4±7.2
50 % EtOH	2.4±0.8	6.6±1.3	1.1±1.5	9.4±1.5	17.9±1.8	4.5±1.4
60 % EtOH	0.3±0.5	7.0±1.6	0	14.6±1.2	14.4±2.2	5.4±1.2
70 % EtOH	0.9±1.2	5.5±1.6	0	16.0±0.5	13.0±0.2	5.3±0.9

[a] The contents of helix, β-sheet and random coil were roughly estimated through an online tool named BeStSel™ (https://bestsel.elte.hu/index.php). The tool is provided by ELTE Eötvös Loránd University, Budapest, Hungray. The tool makes calculation procedure quite easy for the users. The users only need to upload the raw data and fill in experimental settings. The online tool then automatically smooths the raw data and prints the calculating results of secondary structure. The results contain 8 fitted contents of secondary structures including helix (regular and distorted helix), antiparallel β-sheet (left-twisted, relaxed and right-twisted ones), parallel β-sheet, turn and others (like random coil).

Table S3. A list of mechanical properties of SA fibers and reinforced alginate fibers.

Sample	Strain (%)	Stress (MPa)	Modulus (GPa)	Toughness (MJ m^{-3})
Ca^{2+}：				
R=1:1	16.7±0.9	192.2±14.1	5.3±0.4	22.7±1.6
R=2:1	14.2±1.1	198.6±8.4	5.9±0.3	17.3±0.8
R=3:1	11.3±0.3	227.5±5.9	7.8±0.6	18.5±1.3
R=4:1	10.9±0.9	248.2±10.7	9.2±0.7	18.9±1.0
R=5:1	9.7±1.6	295.1±16.7	12.3±0.8	18.2±1.2
Ca^{2+} (R=5:1)：				
30 % EtOH	11.3±0.4	304.0±9.5	11.1±0.7	24.3±1.5
40 % EtOH	12.9±1.1	341.9±11.9	11.3±1.0	28.7±1.5
50 % EtOH	11.2±0.7	410.2±9.5	15.4±0.7	31.3±1.9
60 % EtOH	7.8±1.3	367.7±13.5	15.9±0.7	22.1±1.6
70 % EtOH	5.8±0.6	297.7±37.9	10.4±0.8	9.8±0.8

Table S4. The mechanical property of SA fibers and reinforced alginate fibers when different proportions of high M_w SA was replaced by low M_w SA.

Replaced percentage by 66 kDa	Strain (%)	Stress (MPa)	Modulus (GPa)	Toughness (MJ m^{-3})
100 % for SA fibers	9.6±3.1	224.5±18.8	9.5±0.5	15.7±1.3
100 % for Reinforced fibers	12.8±2.6	276.1±15.5	9.4±0.8	25.3±1.7
70 % for SA fibers	8.1±0.8	250.3±20.9	9.5±1.1	14.0±1.9
70% for Reinforced fibers	12.2±1.4	286.5±22.5	11.1±0.8	23.5±2.4
50 % for SA fibers	9.3±1.4	284.6±6.9	9.8±0.7	17.5±2.5
50 % for Reinforced fibers	12.7±2.7	305.1±31.1	10.6±0.4	27.4±1.8
20 % for SA fibers	9.8±1.8	294.6±12.0	10.5±0.6	20.1±2.1
20 % for Reinforced fibers	13.1±1.4	361.8±18.2	11.0±0.8	32.0±2.6

Table S5. The M_w and yield of SA extracted from brown seaweeds from different locations.

Alginate source	Molecular weight, M_w×10^5 (g mol^{-1})	Yield (%)	Ref.
Sargassum turbinarioides (Madagascar)	5.528	10	[1]
Laminaria digitata (Moroccan)	1.14	51.8, 44.0	[2]
	2.9-3.9	32-34	
Macrocystis pyrifera (Argentine)	2.1-2.3	25-29	[3]
	0.6-1.0	27-29	
Sargassum vulgare (Brazil)	3.30	16.9	[4]
Sargassum dentifolium (Egyptian)	6.1	3.3	
Sargassum asperifolium (Egyptian)	7.3	12.1	[4, 5]
Sargassum latifolium (Egyptian)	4.2	17.2	

References

1. Fenoradosoa, T. A.; Ali, G.; Delattre, C.; Laroche, C.; Petit, E.; Wadouachi, A.; Michaud, P., Extraction and characterization of an alginate from the brown seaweed Sargassum turbinarioides Grunow. *J. Appl. Phycol.* **2009,** 22, (2), 131-137.

2. Fertah, M.; Belfkira, A.; Dahmane, E. m.; Taourirte, M.; Brouillette, F., Extraction and characterization of sodium alginate from Moroccan Laminaria digitata brown seaweed. *Arabian J. Chem.* **2017,** 10, S3707-S3714.

3. Gomez, C. G.; Pérez Lambrecht, M. V.; Lozano, J. E.; Rinaudo, M.; Villar, M. A., Influence of the extraction–purification conditions on final properties of alginates obtained from brown algae (Macrocystis pyrifera). *Int. J. Biol. Macromol.* **2009,** 44, (4), 365-371.

4. Torres, M. R.; Sousa, A. P. A.; Silva Filho, E. A. T.; Melo, D. F.; Feitosa, J. P. A.; de Paula, R. C. M.; Lima, M. G. S., Extraction and physicochemical characterization of Sargassum vulgare alginate from Brazil. *Carbohydr. Res.* **2007,** 342, (14), 2067-2074.

5. Larsen, B.; Salem, D. M. S. A.; Sallam, M. A. E.; Mishrikey, M. M.; Beltagy, A. I., Characterization of the alginates from algae harvested at the Egyptian Red Sea coast. *Carbohydr. Res.* **2003,** 338, (22), 2325-2336.
